# Supplementary material for: The Prognostic Value of Derivatives-Reactive Oxygen Metabolites (d-ROMs) for Cardiovascular Disease Events and Mortality: A Review
Source: Antioxidants (Basel). 2022 Aug 9;11(8):1541. doi: 10.3390/antiox11081541 (PMC9405117; doi:10.3390/antiox11081541)
Supplement: Supplementary file 1 [file antioxidants-11-01541-s001.zip › antioxidants-1796438-supplementary.pdf]

Supplementary materials

BOX1. Systems affecting RedOx homeostasis

ROS sources: ROS can be produced endogenously as a consequence of different physiological processes. ROS are generated during normal cellular metabolism, during inflammation or as a consequence of the innate immune response to infections. The main enzymes involved in ROS production are **mitochondrial enzymes** of the respiratory chain, xanthine oxidase (**XO**), NADP(H) oxidase (**NOX**), endothelial nitric oxide synthetase (**eNOS**), and myeloperoxidase (**MPO**). ROS production can also increase as a consequence of the exposure to chemical and physical factors (such as pollution and ultraviolet light) or unhealthy lifestyle habits (e.g. smoking and unhealthy diet).

Endogenous Sources	Exogenous Sources	Lifestyle Habits
Mitochondria Respiratory Chain	Pollution	Smoking
XO	Ultraviolet Light	Unhealthy Diet
NOX	Ionizing Radiation	Sedentary
eNOS	Drugs	Alcohol Abuse
MPO	Environmental Toxins	Physical activity Overtraining
	Pesticides	Psychological Stress

Antioxidant defences:

Enzymatic antioxidant defences are represented by proteins endowed with catalytic activities. They are characterized by high specificity toward a defined species of ROS. In this class are grouped superoxide dismutase (**SOD**), glutathione peroxidase (**GPx**) and catalase (**CAT**).

Non-enzymatic antioxidant defences are represented by chemical substances, amino acids or peptides that directly or indirectly quench or inhibit the oxidation of endogenous macro-biomolecules (proteins, lipid and nucleic acids). In this class are grouped ascorbic acid (**Vitamin C**), α-tocopherol (**Vitamin E**), glutathione (**GSH**), carotenoids and flavonoids.

Direct antioxidant defences are represented by both enzymatic or non-enzymatic antioxidants able to donate one or more electrons both *in vivo* and *in vitro*. In this class are grouped enzymes (**SOD**, **GPx**, **CAT**) and non-enzymatically active molecules (**Vitamin A**, **Vitamin E**, **Vitamin C**, **GSH**, **carnosine**).

Indirect antioxidant defences are represented by non-enzymatic antioxidants which increase the production of NADH antioxidant equivalents. Therefore, they do not directly quench or inhibit oxidation. They mainly act as co-factors in different metabolic pathways (e.g. Pentose Phosphate Pathway, PPP) or are involved in relevant transformation processes, such as the conversion of homocysteine (pro-oxidant) to cysteine (antioxidant). **Vitamins of the B group** and **polyglucosamine** are good examples of natural indirect antioxidants.[13,16]
